# Comparative evaluation of fracture resistance of re-attached teeth using self-adhesive bioactive flowable composite after preconditioning the fractured coronal fragments with different remineralizing agents

**DOI:** 10.12688/f1000research.134942.1

**Published:** 2023-07-06

**Authors:** Pratik Rathod, Nikhil Mankar

**Affiliations:** 1 Post Graduate Student Department of Conservative Dentistry And Endodontics, Sharad Pawar Dental College, Datta Meghe Institute of Higher Education and Research, wardha, Mahashtra, 442001, India; 2 Associate professor, Department of Conservative Dentistry and Endodontics, Sharad Pawar Dental College, Datta Meghe Institute of Higher Education and Research, Sawangi, wardha, Maharashtra, 442001, India

**Keywords:** Traumatic dental injury, Casein phosphopeptide–amorphous calcium phosphate, Sodium fluoride, Uncomplicated crown fracture, Bioactive, Reattachment technique, Fracture resistance.

## Abstract

One of the common forms of dental injury is anterior crown fractures, which mainly affects the teenagers and young adults. Fractures of the coronal portion of the permanent incisors characterize 18–22% of total traumatic injuries to dental hard tissues; of which 96% of them comprise the maxillary incisors. Uncomplicated fracture of crown is one of the most common types of dental traumatic injury. Dental trauma has an emotional impact on the patient’s overall health and can serious harm to the dentition. The treatment as well as prognosis of the fracture of coronal portion is a major challenge for a dentist because it has to accomplish various parameters like need to obtain an aesthetical result that approaches itself to its natural form and measurement, opaqueness and translucency of the original tooth structure in obtaining a effective restoration. It is suggested that reattachment of the fractured fragment is the best procedure for restoring an uncomplicated fracture of a crown, if a fragment is present. Reattachment of the fractured fragment offers major advantages over the conventional composite restoration. In reattachment, enhanced aesthetics is obtained because enamel’s true shape, colour, intensity, and surface texture are preserved. It re-establishes the major function and produces the favourable emotive and social response from the side of the patient. The reattached fragments are susceptible to further fracture when the restored teeth undergo further trauma. The resistance of the fractured teeth that has been reattached is the subject of the majority of concerns. Preconditioning the fractured fragments with remineralizing agents may aid in hydration. Thus, study will be conducted to evaluate the resistance of the fracture of a tooth that is reattached and pre-treated with remineralizing agents such as sodium fluoride and casein phosphopeptide-amorphous calcium phosphate and further reattached using one of the self-adhesive bioactive composite.

## Introduction

Fracture of the crown of anterior teeth is one of the most common types of dental trauma that primarily affects adolescents and young adults. The location of upper incisors and their pattern of eruption brings a major risk for the dental trauma. ^1^ Dental trauma is more prevalent in case of contact sports, road traffic accidents, outdoor events, and in case of falls. Orofacial trauma comprises about 5% of all injuries to the body, while traumatic dental injuries (TDI) has been reported 15.2% prevalent worldwide. ^2^ With a prevalence of 17% to 48%, fractures of uncomplicated crown are the utmost or frequent types of dental trauma. 18–22% of all traumatic injuries to the hard tissues of dental origin result in fractures of the coronal portion of permanent upper incisors, and 96% of them involves the upper incisors (80% of them are central incisors and 16% of them are lateral incisors). Injuries caused by dental trauma not only affects the dentition, but also have a major impact on psychological status of the patient. According to the International Association of Dental Traumatology reattachment of the fractured fragment is the best method to restore uncomplicated fractures of the crown of permanent teeth, if the fractured fragment is available. ^3^ Many of these problems have been resolved because of the advancement in field of bonded aesthetic dentistry and its ongoing evolution. A better prognosis can result by using intermediate composite material and the lack of further added preparations, which can improve the adhesion of fractured fragments. Although composite restorations of direct and indirect type and prosthesis are other treatment options, but fragment reattachment has been proven to be a preferable alternative for restoring aesthetics, improved functions and natural anatomy of the tooth. In Addition to this, it is cost-effective and time-saving. ^4^ Because the general anatomical shape of the tooth, colour, and texture of the tooth surface are preserved, reattaching the fragment can produce pleasing aesthetics that last for a long time. This procedure is rather simple and fairly conservative. It restores tooth function and encourages the patient to feel better immediately on an emotional and social level. ^5^ If another traumatic incident occurs or when the restored teeth are used in a way that is not physiological, the reattached fragments are vulnerable to breaking again. The resistance of the fractured teeth that has been reattached is the subject of the majority of concerns. The significant factor is whether the fragment of fractured tooth and the residual tooth can form a stable, predictable union. Activa bioactive has rubberized resin component having more fracture resistance and shock absorption and being a smart material adapts to PH cycle. It has all the desired properties such as improved hydrophilicity, sealing ability, bond strength. The conventional flowable composite clinically used in dentistry for reattachment varies in terms of bond strength and adhesion therefore newer materials with improved properties are desired and henceforth in this study the material Activa bioactive will be tested. The time interval between the traumatic injury and reattachment as well as the hydration of the broken fragment, which preserves the vitality and original shine of that of the natural tooth, are the major factors in the achievement of re attachment of fractured fragments. Dentin moisture is necessary to fortify the binding between composite resin and dentin. According to findings from the literature, hydrated fractured tooth fragments have a stronger bond than dehydrated pieces. ^6^ For preconditioning of the fractured fragments use of remineralizing agents not only may aid in hydration but also for up taking maximum of the mineral ions. But, the study about the result of remineralizing agents affecting the bond strength of re attached tooth is limited. Henceforth, a study will be done to assess the fracture resistance of the teeth that is reattached using self- adhesive bioactive composite and were pre-treated with remineralizing agents such as sodium fluoride, casein phosphopeptide–amorphous calcium phosphate (CPP-ACP), prior to re-attachment.

## Objectives

1) To evaluate fracture resistance of reattached fractured tooth preconditioning with CPP-ACP luted with self- adhesive bioactive material using universal testing machine.
2) To evaluate fracture resistance of reattached fractured tooth preconditioning with NaF as preconditioning agent luted self-adhesive bioactive material using universal testing machine.
3) To compare fracture resistance of reattached fractured tooth with CPP-ACP and NaF as preconditioning agents luted with self-adhesive bioactive material using universal testing machine.

## Methods

### Trial design

In-vitro study.

### Materials required

1. Casein Phosphopeptide – Amorphous Calcium Phosphate (CPP-ACP), (GC tooth Mousse, GC India)
2. 2% Sodium Fluoride (2% NAF) (SEPTODONT Sodium Fluoride Gel -Flucol Gel)
3. Self-adhesive Bioactive flowable composite material – (ACTIVA bioactive-CEMENT, Pulpdent)
4. Etchant -37% phosphoric acid (Prime etching liquid, India)
5. Adhesive agent (Adper Single Bond 2 Adhesive – 3M ESPE)

### Inclusion criteria

1. Sound upper central incisors removed due to periodontal reasons.
2. Teeth devoid of restorations.
3. Non carious teeth.

### Exclusion criteria

1. Teeth with previous root canal treatment.
2. Extensively carious tooth.
3. Abrasion, attrition, fluorosis, or other enamel defects.
4. Teeth with developmental anomalies.
5. Teeth with external and internal resorption.

### Intervention

➢ Total of 50 samples will be considered in this study.
➢ Samples will be divided into 2 groups, 25 in each group corresponding to the 1 and 2 sample sets.
➢ Group 1: Casein phosphopeptide-amorphous calcium phosphate as preconditioning agent.
➢ Group 2: Sodium fluoride as preconditioning agent.

### Procedure

For the study, a total of 50 freshly extracted, sound permanent human maxillary central incisor teeth will be used.

***Sectioning of the teeth***

To simulate an uncomplicated crown fracture, extracted teeth will be sectioned at incisor third of crown using low speed double sided diamond disk. The direction of diamond disk will be in perpendicular direction to the long axis of the tooth.

***Preconditioning of teeth***

Sectioned fragments will be immersed in agents that are re mineralizable such as sodium fluoride and CPP-ACP for a predetermined contact period i.e., 30 min. Then, the fragments from the coronal portion will be attached to the residual structures of tooth with self-adhesive flowable bioactive composite material.

***Re-attachment of fragments***

The fragments of the fractured teeth that are preconditioned and the tooth structure that is residual will be rinsed thoroughly with distilled water. After thorough rinsing, acid etching will be done. Application of etchant i.e., 37% phosphoric acid (Prime etching liquid, india) for 15 sec will be carried out, followed by rinsing of the tooth with water for 15 s. After that, both surfaces will be dried with air at maximum of 5 seconds in order to keep the surface moist. The process of applying adhesive bonding agent to the surfaces will be carried out after acid etching. The first coat of adhesive agent (Adper Single Bond 2 Adhesive – 3M ESPE) will be applied to the surfaces that are sectioned for 10 s, and then, second layer of adhesive agent will be applied. Then coats will be cured by light for maximum of 20 s after being air-thinned to remove any surplus. The fractured fragments will be restored together, reattached, and curing is done for 20 seconds on the labial surface and palatal surface respectively using self-adhesive bioactive composite material (ACTIVA BioACTIVE-CEMENT, Pulpdent). The specimens will be placed in artificial saliva after reattachment and left there until their fracture resistance is assessed.

***Evaluation of fracture resistance of teeth***

The tooth samples will be numbered for easy identification and embedded in blocks of cold-cure acrylic resin (2 cm × 2 cm) (DPI RR cold cure, India) up to the cingulum for evaluation of fracture resistance. The universal testing machine will be used to evaluate the fracture resistance.

The force exerted by the applicator tip will be placed exactly perpendicular to the line of fracture in relation to the surface of crown labially having the speed of crosshead at 1 mm/min.

And having cell load of 500 newton. Force that is needed to fracture the tooth will be assessed in unit that is Newton.

### Sample size calculation

Formula Using Mean difference on fracture resistance (FR) in N (newton)

$$n1=n2=2\frac{{\left(Z_{\alpha }+Z_{\beta }\right)}^{2}\sigma^{2}}{{\left(\delta \right)}^{2}}$$

$$Z_{\alpha }=1.96$$

α=Type I error at5%

Z _β_ = 0.84 at 5% type I error.

σ=std.dev

Primary Variable fracture resistance (FR)

((Fracture resistance (FR) in reattached teeth preconditioned with CPP-ACP group) Mean ± SD. = 215.5 ± 81.16 As per Reference article)

((Fracture resistance (FR) in reattached teeth preconditioned with NaF group) Mean ± SD. = 141.29 ± 54.25 As per Reference article)

$$\text{Difference}=\left(215.58-141.29\right)=74.29$$

Std.dev=81.16

As per reference articles.

$$\text{N1}=2^{*}\left[{\left(1.84+0.84\right)}^{2}{\left(81.16\right)}^{2}\right]/{\left(74.29\right)}^{2}=25$$

Total samples required = 25 per group

Formula Reference: 6

For the ease of calculation and statistics, the sample size confirmed to 50 consisting of 25 subjects in each group.

### Statistical method

All the results will be calculated using SPSS version 27 software. All the demographic data variable assessment will be done for quantitative assessment in mean std dev minimum & maximum & in frequency & percentage for qualitative assessment. Data for outcomes variables will be tested for normality using kalmogorov-smirnov. The comparative analysis of the fracture resistance will be evaluated on the measurement of Newton. Unpaired t test will be used to find the significant difference between the mean of the 2 groups. P-value ≤ 0.05 will be considered as significant at 5% level of significance and 95% confidence of interval.

### Outcomes

It is expected that preconditioning of fragment with sodium fluoride may give better results as compared to Casein Phosphopeptide–Amorphous Calcium Phosphate on fracture resistance of reattached fragment with self-adhesive bioactive flowable composite.

### Dissemination

The focus of this study is to assess the fracture resistance of the reattached teeth preconditioned with various remineralizing agents. Preconditioning of fragment may further contribute to the increased fracture resistance of reattached fractured tooth.

### Ethical considerations

Ethical approval was received from Datta Meghe Institute of Higher Education and Research, Sawangi, Wardha (Maharashtra), India.

IEC reference number - DMIHER (DU)/IEC/2023/582.

Written informed consent will be taken from patients who undergo extraction regarding the use of extracted teeth for the study purpose.

### Study status

Not started yet.

## Discussion

The task is to handle the tooth with utmost care that further lessen the damage of the teeth with coronal fractures of upper incisors. Several restorative techniques of direct and indirect type are in use for the treatment of fractured teeth, although such techniques often compromise a lot of the natural healthy tooth structure. ^7^ If there is only a slight violation in the area of the biological width and a complete fragment of the fractured tooth is available, the re attachment of the fractured fragment method can be considered. Bruschi-Alonso *et al.* stated that the first choice of treatment for fractured tooth having uncomplicated crown fracture should be the re-attachment of fractured fragment. ^8^ Reattachment offers many benefits over other procedures as it is a minimal invasive in nature, uncomplicated, and cost-effective process. It aids in maintaining enduring aesthetics and is highly accepted by the patient.

Hydrating fragments of fracture play a very important role in improvement of the resistance of fracture at re-attachment interface. ^4^^,^^9^ Time interval during or after trauma, media that is used for storage, and dry time before the re-attachment of the fractured fragments are crucial aspects and they impact the resistance of fracture and adhesive strength of re-attached fragments. Most commonly affected teeth by dental traumatic injury are maxillary central incisors. Hence maxillary central incisors are selected for the study. In regard to the method used, the fragments of tooth will be attained by segmenting with a diamond disk in spite of fracturing. Badami *et al.* and Reis *et al.* stated that the tooth surface that is sectioned is totally different from that of a tooth surface that is fractured. ^10^ The path of the fractured line in case of a tooth that is sectioned is indicated by the position of the disk, it inclines to run in a direction parallel to the direction of prisms of enamel in a region of fragmentation. In a while, because trauma has a nature of not proceeding linearly or with good adjustment, this direction may not correctly depict the accurate circumstances of the trauma. Nevertheless, the standardisation of the fragmented fracture that is necessary to lessen the bias that is of confounding nature was made possible by simulating the fracture by use of a cutting disk. According to Garcia *et al.* and de Souza *et al.*, fractured fragment is reattached by a technique of no preparation and a bonding system having intermediate resin composite comprising good mechanical properties that have the capacity to restore part of the resistance of the tooth that is fracture. ^11^ Preconditioning of fractured fragments with remineralizing agents may aid in hydration. Thus, study will be conducted to assess the resistance of fracture of teeth that is reattached and are pre-treated with various remineralizing agents such as sodium fluoride and casein phosphopeptide-amorphous calcium phosphate and reattached using self-adhesive bioactive composite.

## Data Availability

Not applicable as this is a protocol.
