# Comparative evaluation of fracture resistance of re-attached teeth using self-adhesive bioactive flowable composite after preconditioning the fractured coronal fragments with different remineralizing agents

**DOI:** 10.12688/f1000research.134942.3

**Published:** 2025-03-11

**Authors:** Pratik Rathod, Nikhil Mankar

**Affiliations:** 1 Post Graduate Student Department of Conservative Dentistry And Endodontics, Sharad Pawar Dental College, Datta Meghe Institute of Higher Education and Research, wardha, Mahashtra, 442001, India; 2 Associate professor, Department of Conservative Dentistry and Endodontics, Sharad Pawar Dental College, Datta Meghe Institute of Higher Education and Research, Sawangi, wardha, Maharashtra, 442001, India

**Keywords:** Traumatic dental injury, Casein phosphopeptide–amorphous calcium phosphate, Sodium fluoride, Uncomplicated crown fracture, Bioactive, Reattachment technique, Fracture resistance.

## Abstract

One of the common forms of dental injury is anterior crown fractures, which mainly affect teenagers and young adults. Fractures of the coronal portion of the permanent incisors characterize 18–22% of total traumatic injuries to dental hard tissues, of which 96% of them comprise the maxillary incisors. An uncomplicated fracture of the crown is one of the most common types of dental traumatic injury. Dental trauma has an emotional impact on the patient’s overall health and can seriously harm to the dentition. The treatment, as well as prognosis of the fracture of the coronal portion, is a major challenge for a dentist because it has to accomplish various parameters like the need to obtain an aesthetical result that approaches itself to its natural form and measurement, opaqueness and translucency of the original tooth structure in obtaining an effective restoration. It is suggested that reattachment of the fractured fragment is the best procedure for restoring an uncomplicated crown fracture, if a fragment is present. Fragment reattachment offers superior aesthetics and function compared to composite restorations, preserving the tooth’s natural characteristics, like the tooth’s true shape, colour, intensity, and surface texture. Success depends on the fracture line’s direction and location; simple enamel or enamel-dentin fractures are ideal. However, reattached fragments remain vulnerable to subsequent trauma. The reattached fragments are susceptible to further fracture when the restored teeth undergo further trauma. The resistance of the fractured teeth that have been reattached is the subject of the majority of concerns. Preconditioning the fractured fragments with remineralizing agents may aid in hydration. Thus, a study will be conducted to evaluate the resistance of the fracture of a tooth that is reattached and pre-treated with remineralizing agents such as sodium fluoride and casein phosphopeptide-amorphous calcium phosphate and further reattached using one of the self-adhesive bioactive composite.

## Introduction

Fracture of the crown of anterior teeth is one of the most common types of dental trauma that primarily affects adolescents and young adults. The location of upper incisors and their pattern of eruption brings a major risk for the dental trauma. ^1^ Dental trauma is more prevalent in case of contact sports, road traffic accidents, outdoor events, and in case of falls. Orofacial trauma comprises about 5% of all injuries to the body, while traumatic dental injuries (TDI) has been reported 15.2% prevalent worldwide. ^2^ With a prevalence of 17% to 48%, fractures of uncomplicated crown are the utmost or frequent types of dental trauma. 18–22% of all traumatic injuries to the hard tissues of dental origin result in fractures of the coronal portion of permanent upper incisors, and 96% of them involves the upper incisors (80% of them are central incisors and 16% of them are lateral incisors). Injuries caused by dental trauma not only affects the dentition, but also have a major impact on psychological status of the patient. According to the International Association of Dental Traumatology reattachment of the fractured fragment is the best method to restore uncomplicated fractures of the crown of permanent teeth, if the fractured fragment is available. ^3^ Many of these problems have been resolved because of the advancement in field of bonded aesthetic dentistry and its ongoing evolution. A better prognosis can result by using intermediate composite material and the lack of further added preparations, which can improve the adhesion of fractured fragments. Although composite restorations of direct and indirect type and prosthesis are other treatment options, but fragment reattachment has been proven to be a preferable alternative for restoring aesthetics, improved functions and natural anatomy of the tooth. In Addition to this, it is cost-effective and time-saving. ^4^ Because the general anatomical shape of the tooth, colour, and texture of the tooth surface are preserved, reattaching the fragment can produce pleasing aesthetics that last for a long time. This procedure is rather simple and fairly conservative. It restores tooth function and encourages the patient to feel better immediately on an emotional and social level. ^5^ If another traumatic incident occurs or when the restored teeth are used in a way that is not physiological, the reattached fragments are vulnerable to breaking again. The resistance of the fractured teeth that has been reattached is the subject of the majority of concerns. The significant factor is whether the fragment of fractured tooth and the residual tooth can form a stable, predictable union. Activa bioactive has rubberized resin component having more fracture resistance and shock absorption and being a smart material adapts to PH cycle. It has all the desired properties such as improved hydrophilicity, sealing ability, bond strength. Activa Bioactive Flowable Composite is designed for a broad range of restorative applications, leveraging its unique combination of bioactivity and flowability. Its clinical indications stem from its ability to release calcium, phosphate, and fluoride ions, promoting remineralization and reducing secondary caries risk, making it particularly suitable for high caries risk patients, deep cavities, and those with xerostomia. The flowable consistency facilitates use in various cavity classifications (I-V), pit and fissure sealants, liners/bases, and repair of enamel or existing restorations, aligning with minimally invasive dentistry principles. Its moisture tolerance and shock-absorbing properties further expand its utility, especially in geriatric and pediatric dentistry, and in situations where traditional resin materials might be contraindicated due to allergies or biocompatibility concerns. Additionally, its dual-cure nature and ability to bond to tooth structure make it suitable for fracture reattachment, providing a bioactive and durable repair. The conventional flowable composite clinically used in dentistry for reattachment varies in terms of bond strength and adhesion therefore newer materials with improved properties are desired and henceforth in this study the material Activa bioactive will be tested. The hydration status of the fragment significantly influences the successful reattachment of a fractured tooth fragment. Dehydrated fragments exhibit decreased fracture strength and may discolour. Studies demonstrate a wide range of fracture resistance in reattached teeth, with hydration emerging as a crucial factor. Rehydrating fragments before reattachment significantly improves fracture resistance, as evidenced by increased strength in both hydrated and rehydrated groups compared to dehydrated ones. Hydration prevents collagen fibre collapse in the dentin, enhances bond strength, and maintains aesthetic appeal by minimizing discolouration. The time interval between the traumatic injury and reattachment as well as the hydration of the broken fragment, which preserves the vitality and original shine of that of the natural tooth, are the major factors in the achievement of re attachment of fractured fragments. Dentin moisture is necessary to fortify the binding between composite resin and dentin. According to findings from the literature, hydrated fractured tooth fragments have a stronger bond than dehydrated pieces. ^6^ For preconditioning of the fractured fragments use of remineralizing agents not only may aid in hydration but also for up taking maximum of the mineral ions like calcium and phosphate. But, the study about the result of remineralizing agents affecting the bond strength of re attached tooth is limited. Henceforth, a study will be done to assess the fracture resistance of the teeth that is reattached using self- adhesive bioactive composite and were pre-treated with remineralizing agents such as sodium fluoride, casein phosphopeptide–amorphous calcium phosphate (CPP-ACP), prior to re-attachment.

## Objectives

1) To evaluate the fracture resistance of reattached fractured teeth preconditioned with CPP-ACP and luted with self-adhesive bioactive material, using a universal testing machine.
2) To evaluate the fracture resistance of reattached fractured teeth preconditioned with NaF and luted with self-adhesive bioactive material, using a universal testing machine.
3) To compare the fracture resistance of reattached fractured teeth preconditioned with CPP-ACP and NaF, and luted with self-adhesive bioactive material, using a universal testing machine.

## Methods

### Trial design

In-vitro study.

### Materials required

1. Casein Phosphopeptide – Amorphous Calcium Phosphate (CPP-ACP), (GC tooth Mousse, GC India)
2. 2% Sodium Fluoride (2% NAF) (SEPTODONT Sodium Fluoride Gel -Flucol Gel)
3. Self-adhesive Bioactive flowable composite material – (ACTIVA bioactive-CEMENT, Pulpdent)
4. Etchant -37% phosphoric acid (Prime etching liquid, India)
5. Adhesive agent (Adper Single Bond 2 Adhesive – 3M ESPE)
6. Artificial saliva

### Inclusion criteria

1. Sound upper central incisors removed due to periodontal reasons.
2. Teeth devoid of restorations.
3. Non carious teeth.

### Exclusion criteria

1. Teeth with previous root canal treatment.
2. Extensively carious tooth.
3. Abrasion, attrition, fluorosis, or other enamel defects.
4. Teeth with developmental anomalies.
5. Teeth with external and internal resorption.

### Intervention

➢ Total of 50 samples will be considered in this study.
➢ Samples will be divided into 2 groups, 25 in each group corresponding to the 1 and 2 sample sets.
➢ Group 1: Casein phosphopeptide-amorphous calcium phosphate as preconditioning agent.
➢ Group 2: Sodium fluoride as preconditioning agent.

### Procedure

For the study, a total of 50 freshly extracted, sound permanent human maxillary central incisor teeth will be used.

***Sectioning of the teeth***

To simulate an uncomplicated crown fracture, extracted teeth will be sectioned at incisor third of crown using low speed double sided diamond disk. The direction of diamond disk will be in perpendicular direction to the long axis of the tooth.

***Preconditioning of teeth***

Sectioned fragments will be immersed in agents that are re mineralizable such as sodium fluoride and CPP-ACP for a predetermined contact period i.e., 30 min. Then, the fragments from the coronal portion will be attached to the residual structures of tooth with self-adhesive flowable bioactive composite material.

***Re-attachment of fragments***

The fragments of the fractured teeth that are preconditioned and the tooth structure that is residual will be rinsed thoroughly with distilled water. After thorough rinsing, acid etching will be done. Application of etchant i.e., 37% phosphoric acid (Prime etching liquid, india) for 15 sec will be carried out, followed by rinsing of the tooth with water for 15 s. ^7^ After that, both surfaces will be dried with air at maximum of 5 seconds in order to keep the surface moist. The process of applying adhesive bonding agent to the surfaces will be carried out after acid etching. The first coat of adhesive agent (Adper Single Bond 2 Adhesive – 3M ESPE) will be applied to the surfaces that are sectioned for 10 s, and then, second layer of adhesive agent will be applied. Then coats will be cured by light for maximum of 20 s after being air-thinned to remove any surplus. ^8^ The fractured fragments will be restored together, reattached, and curing is done for 20 seconds on the labial surface and palatal surface respectively using self-adhesive bioactive composite material (ACTIVA BioACTIVE-CEMENT, Pulpdent). ACTIVA BioACTIVE Flowable Composite will be applied to both fractured surfaces—the fragment and the remaining tooth structure—using the manufacturer’s delivery tip. This will ensure even distribution across the sectioned areas, facilitating optimal adaptation and bonding during the reattachment process. The specimens will be placed in artificial saliva after reattachment and left there until their fracture resistance is assessed.

***Evaluation of fracture resistance of teeth***

The tooth samples, numbered for easy identification, will be embedded in blocks of cold-cure acrylic resin (2 cm × 2 cm) (DPI RR cold cure, India) up to the cingulum for evaluation of fracture resistance. A universal testing machine will be utilized to assess the fracture resistance. The force exerted by a chisel-shaped crosshead with a 1 mm edge will be applied exactly perpendicular to the line of fracture in relation to the labial surface of the crown. The crosshead speed will be set at 1 mm/min, and the cell load will be 500 Newtons. The force required to fracture the tooth will be measured and recorded in Newtons.

### Sample size calculation

Formula Using Mean difference on fracture resistance (FR) in N (newton)

$$n1=n2=2\frac{{\left(Z_{\alpha }+Z_{\beta }\right)}^{2}\sigma^{2}}{{\left(\delta \right)}^{2}}$$

$$Z_{\alpha }=1.96$$

α=Type I error at5%

Z _β_ = 0.84 at 5% type I error.

σ=std.dev

Primary Variable fracture resistance (FR)

((Fracture resistance (FR) in reattached teeth preconditioned with CPP-ACP group) Mean ± SD. = 215.5 ± 81.16 As per Reference article)

((Fracture resistance (FR) in reattached teeth preconditioned with NaF group) Mean ± SD. = 141.29 ± 54.25 As per Reference article)

$$\text{Difference}=\left(215.58-141.29\right)=74.29$$

Std.dev=81.16

As per reference articles.

$$\text{N1}=2^{*}\left[{\left(1.84+0.84\right)}^{2}{\left(81.16\right)}^{2}\right]/{\left(74.29\right)}^{2}=25$$

Total samples required = 25 per group

Formula Reference: 6

For the ease of calculation and statistics, the sample size confirmed to 50 consisting of 25 subjects in each group.

### Statistical method

All the results will be calculated using SPSS version 27 software. All the demographic data variable assessment will be done for quantitative assessment in mean std dev minimum & maximum & in frequency & percentage for qualitative assessment. Data for outcomes variables will be tested for normality using kalmogorov-smirnov. The comparative analysis of the fracture resistance will be evaluated on the measurement of Newton. Unpaired t test will be used to find the significant difference between the mean of the 2 groups. P-value ≤ 0.05 will be considered as significant at 5% level of significance and 95% confidence of interval.

### Outcomes

It is expected that preconditioning of the fragment with either sodium fluoride or Casein Phosphopeptide–Amorphous Calcium Phosphate will yield comparable results in terms of fracture resistance of the reattached fragment when using a self-adhesive bioactive flowable composite.

### Dissemination

The focus of this study is to assess the fracture resistance of the reattached teeth preconditioned with various remineralizing agents. Preconditioning of fragment may further contribute to the increased fracture resistance of reattached fractured tooth.

### Ethical considerations

Ethical approval was received from Datta Meghe Institute of Higher Education and Research, Sawangi, Wardha (Maharashtra), India.

IEC reference number - DMIHER (DU)/IEC/2023/582.

Written informed consent will be taken from patients who undergo extraction regarding the use of extracted teeth for the study purpose.

### Study status

Not started yet.

## Discussion

The task is to handle the tooth with utmost care that further lessen the damage of the teeth with coronal fractures of upper incisors. Several restorative techniques of direct and indirect type are in use for the treatment of fractured teeth, although such techniques often compromise a lot of the natural healthy tooth structure. ^9^ If there is only a slight violation in the area of the biological width and a complete fragment of the fractured tooth is available, the re attachment of the fractured fragment method can be considered. Bruschi-Alonso *et al.* stated that the first choice of treatment for fractured tooth having uncomplicated crown fracture should be the re-attachment of fractured fragment. ^10^ Reattachment offers many benefits over other procedures as it is a minimal invasive in nature, uncomplicated, and cost-effective process. It aids in maintaining enduring aesthetics and is highly accepted by the patient.

Hydrating fragments of fracture play a significant role in improvement of the resistance of fracture at re-attachment interface. ^4^^,^^11^ Time interval during or after trauma, media that is used for storage, and dry time before the re-attachment of the fractured fragments are crucial aspects and they impact the resistance of fracture and adhesive strength of re-attached fragments. Most commonly affected teeth by dental traumatic injury are maxillary central incisors. Hence maxillary central incisors are selected for the study. In regard to the method used, the fragments of tooth will be attained by segmenting with a diamond disk in spite of fracturing. Instead of fracturing the teeth, fragments will be carefully sectioned with a diamond disk to ensure a controlled and reproducible fracture surface for the study. Badami *et al.* and Reis *et al.* stated that the tooth surface that is sectioned is totally different from that of a tooth surface that is fractured. ^12^ The path of the fractured line in case of a tooth that is sectioned is indicated by the position of the disk, it inclines to run in a direction parallel to the direction of prisms of enamel in a region of fragmentation. In a while, because trauma has a nature of not proceeding linearly or with good adjustment, this direction may not correctly depict the accurate circumstances of the trauma. To minimize confounding bias and ensure standardization of the fractured surfaces, a diamond disk will be used to simulate the fracture, thereby achieving consistent fragmentation. According to Garcia *et al.* and de Souza *et al.*, fractured fragment is reattached by a technique of no preparation and a bonding system having intermediate resin composite comprising good mechanical properties that have the capacity to restore part of the resistance of the tooth that is fracture. ^13^ Preconditioning of fractured fragments with remineralizing agents may aid in hydration. Thus, this study will evaluate the fracture resistance of reattached teeth pretreated with sodium fluoride or casein phosphopeptide-amorphous calcium phosphate and bonded with a self-adhesive bioactive composite.

## Data Availability

Not applicable as this is a protocol.
