# Supplementary material for: Comparative evaluation of fracture resistance of re-attached teeth using self-adhesive bioactive flowable composite after preconditioning the fractured coronal fragments with different remineralizing agents
Source: F1000Res. 2025 Mar 11;12:791. Originally published 2023 Jul 6. [Version 3] doi: 10.12688/f1000research.134942.3 (PMC12405839; doi:10.12688/f1000research.134942.3)
Supplement: Peer review history

# Reviewer report: Reviewer response for version 3

**DOI:** 10.5256/f1000research.179080.r406848

**Published:** 2025-09-02

**Authors:** Pankaj Dhawan

**Affiliations:** 1 Manav Rachna International Institute of Research and Studies, Faridabad, Haryana, India

1] While the comparison between the two experimental groups is well-founded, the study design lacks a **control group** (e.g., reattachment with no preconditioning agent). A control group would have allowed for the quantification of the absolute benefit of preconditioning itself, rather than just the relative performance of the two agents. However, for the specific comparative objective stated by the authors, the current design is acceptable.

 2]The protocol states that specimens will be stored in artificial saliva "until their fracture resistance is assessed". This period should be standardised and explicitly stated (e.g., 24 hours, 7 days) to ensure consistent water sorption and material maturation across all samples.

 3]The composition of the artificial saliva and the storage temperature (e.g., 37°C in an incubator) should be detailed.

 4]The specific model and, more importantly, the power output/irradiance (in mW/cm2) of the light-curing unit used to cure the adhesive and composite are not mentioned. This is a critical parameter that directly influences the degree of conversion and the final mechanical properties of the resin-based materials.

 Accept with above mentioned changes.

Is the study design appropriate for the research question?

Yes

Is the rationale for, and objectives of, the study clearly described?

Yes

Are sufficient details of the methods provided to allow replication by others?

Partly

Are the datasets clearly presented in a useable and accessible format?

Yes

Reviewer Expertise:

Dentistry

I confirm that I have read this submission and believe that I have an appropriate level of expertise to confirm that it is of an acceptable scientific standard.

# Reviewer report: Reviewer response for version 3

**DOI:** 10.5256/f1000research.179080.r370473

**Published:** 2025-03-18

**Authors:** Srinidhi Surya Raghavendra

**Affiliations:** 1 Conservative Dentistry & Endod, Dr DY Patil Vidyapeeth University Dr DY Patil Dental College and Hospital, Pune, Maharashtra, India

The concerns I had and the corrections I have suggested have been done by the authors satisfactorily.

Is the study design appropriate for the research question?

Yes

Is the rationale for, and objectives of, the study clearly described?

Yes

Are sufficient details of the methods provided to allow replication by others?

Partly

Are the datasets clearly presented in a useable and accessible format?

Partly

Reviewer Expertise:

Esthetics and restorative materials and techniques, Bonding to dentin, nanoleakage in dentin

I confirm that I have read this submission and believe that I have an appropriate level of expertise to confirm that it is of an acceptable scientific standard.

# Reviewer report: Reviewer response for version 2

**DOI:** 10.5256/f1000research.161550.r291030

**Published:** 2024-06-14

**Authors:** Srinidhi Surya Raghavendra

**Affiliations:** 1 Conservative Dentistry & Endod, Dr DY Patil Vidyapeeth University Dr DY Patil Dental College and Hospital, Pune, Maharashtra, India

Abstract: 1) One of the parameters for selecting the reattachment of the fragment as a treatment option is the direction and location of the fracture line. This point needs to be mentioned.

 Introduction:

 1) Give brief background about Activa's clinical indications.

 2) One sentence as to how hydration helps in increasing bond strength is needed.

 3) In the sentence 'For preconditioning of the fractured fragments use of remineralizing agents not only may aid in hydration but also for up taking maximum of the mineral ions', what are the ions we are talking about?

 4) The last sentence needs to be re phrased.

 Objectives: The points 1 and 2 can be written as 'To evaluate the fracture resistance of reattached tooth fragment preconditioned with CPP-ACP or NAF and luted with self adhesive bioactive material'. The 3rd point can be modified similarly.

 Method:

 1) How is the Activa placed on the sectioned areas? Is it equally placed on both with the delivery tip?

 2) There is no mention of artificial saliva in the materials required.

 3) After how much time is the fracture resistance assessed?

 4) In the line 'The force exerted by the applicator tip will be placed exactly perpendicular', what is the shape & size of the load applying tip?

 Outcome:

 How is the statement given valid before the study is conducted? 

 Discussion: 

 1) Re frame this sentence 'In regard to the method used, the fragments of tooth will be attained by segmenting with a diamond disk in spite of fracturing'.

 2) 'lessen the bias that is of confounding nature' - write this so that the whole sentence is easy to understand.

 3) In the sentence 'study will be conducted to assess the resistance of fracture of teeth', it should be resistance **to fracture of teeth'.**

Is the study design appropriate for the research question?

Yes

Is the rationale for, and objectives of, the study clearly described?

Yes

Are sufficient details of the methods provided to allow replication by others?

Partly

Are the datasets clearly presented in a useable and accessible format?

Partly

Reviewer Expertise:

Esthetics and restorative materials and techniques, Bonding to dentin, nanoleakage in dentin

I confirm that I have read this submission and believe that I have an appropriate level of expertise to confirm that it is of an acceptable scientific standard, however I have significant reservations, as outlined above.

# Response

**Published:** 2025-03-06

**Authors:** Pratik Rathod

**Affiliations:** Conservative dentistry, Sharad Pawar Dental College, Datta Meghe institute of higher education and research, wardha, maharashtra, India

Respected Sir,

 I have made all the suggested corrections by reviewers.

 Kindly please review my article.

 Thank you.

# Reviewer report: Reviewer response for version 1

**DOI:** 10.5256/f1000research.148038.r211913

**Published:** 2023-11-27

**Authors:** Anand Marya

**Affiliations:** 1 Department of Orthodontics, Faculty of Dentistry, University of Puthisastra, Phnom Penh, Phnom Penh, Cambodia; 2 Center for Transdisciplinary Research, Saveetha Dental College, Saveetha Institute of Medical and Technical Science, Saveetha University, Chennai, India

Thank you for allowing me to review this paper. I do have the following comments:

1. How was the sample size calculation done? What is the power of the sample?
2. Is there any control group with no remineralizing agent?
3. Please cite references for the methodology? eg. duration of etching etc.

Is the study design appropriate for the research question?

Yes

Is the rationale for, and objectives of, the study clearly described?

Yes

Are sufficient details of the methods provided to allow replication by others?

Yes

Are the datasets clearly presented in a useable and accessible format?

Yes

Reviewer Expertise:

Orthodontics, remineralization, adhesives

I confirm that I have read this submission and believe that I have an appropriate level of expertise to confirm that it is of an acceptable scientific standard, however I have significant reservations, as outlined above.

# Response

**Published:** 2024-01-17

**Authors:** Pratik Rathod

**Affiliations:** Conservative dentistry, Sharad Pawar Dental College, Datta Meghe institute of higher education and research, wardha, maharashtra, India

Thank you, respected sir, for reviewing this paper.

 I have made all the suggested corrections as follows.

**1.** Sample size calculation is done, taking fracture resistance as a variable, keeping the power of sample 80 and the Difference between two groups 74.29.

**2.** sir, there is no control group with remineralizing agents.

**3.** I have added references for the methodology.
